# Access to specialty healthcare in urban versus rural US populations: a systematic literature review

**DOI:** 10.1186/s12913-019-4815-5

**Published:** 2019-12-18

**Authors:** Melissa E. Cyr, Anna G. Etchin, Barbara J. Guthrie, James C. Benneyan

**Affiliations:** 1 School of Nursing, Northeastern University, 360 Huntington Avenue, Boston, MA 02115 USA; 2 VA Boston Healthcare System, 150 South Huntington Avenue, Jamaica Plain, MA 02130 USA; 3 Bouvé College of Health Sciences, Northeastern University, 360 Huntington Avenue, Boston, MA 02115 USA; 4 Healthcare Systems Engineering Institute, Northeastern University, 360 Huntington Avenue, Boston, MA 02115 USA

**Keywords:** Health services accessibility, Rural care, Systematic literature review, Conceptual framework

## Abstract

**Background:**

Access to healthcare is a poorly defined construct, with insufficient understanding of differences in facilitators and barriers between US urban versus rural specialty care. We summarize recent literature and expand upon a prior conceptual access framework, adapted here specifically to urban and rural specialty care.

**Methods:**

A systematic review was conducted of literature within the CINAHL, Medline, PubMed, PsycInfo, and ProQuest Social Sciences databases published between January 2013 and August 2018. Search terms targeted peer-reviewed academic publications pertinent to access to US urban or rural specialty healthcare. Exclusion criteria produced 67 articles. Findings were organized into an existing ten-dimension care access conceptual framework where possible, with additional topics grouped thematically into supplemental dimensions.

**Results:**

Despite geographic and demographic differences, many access facilitators and barriers were common to both populations; only three dimensions did not contain literature addressing both urban and rural populations. The most commonly represented dimensions were *availability and accommodation*, *appropriateness*, and *ability to perceive.* Four new identified dimensions were: *government and insurance policy*, *health organization and operations influence*, *stigma*, and *primary care and specialist influence*.

**Conclusions:**

While findings generally align with a preexisting framework, they also suggest several additional themes important to urban versus rural specialty care access.

## Background

Long delays or complete inaccessibility to primary and specialty care are common across the United States (US) [1, 2]. Elderly, women, children, racial and ethnic minorities, socioeconomically disadvantaged, and individuals with chronic health conditions disproportionately experience greater specialty care access challenges and poorer health outcomes despite geographic residence [3–7], especially in medically underserved urban and rural areas [8, 9]. To reduce disparities, numerous national agencies advocate improved effectiveness in providing essential services among at-risk groups [5, 10]. While conceptual frameworks exist to guide these efforts [11, 12], none specifically focus on US urban versus rural specialty care.

Although there is no universal definition of urban and rural geographic areas, the Rural-Urban Commuting-Area (RUCA) is a common taxonomy that combines work-commuting data with US Census Bureau tracts or zip codes, with 33 categories that range from urbanized to isolated small rural areas [13]. To simplify this continuum, often fewer urban, suburban, and rural classifications are used to identify unique characteristics and specialty access challenges. While the US is predominantly rural, only roughly one-fifth of the total population reside and one-tenth of clinicians practice in these areas [9]. Rural dwellers overall are older, more likely to be veterans or uninsured, and less likely to have completed higher education; in contrast, urban dwellers have increased poverty rates, are more likely to be foreign born, and are less likely to own their home [14]. Urban areas have the highest reported infant mortality rates, homicides, adult major depressive episodes, and mortality from unintended injuries; however, smoking, obesity rates, inactivity levels, suicide, serious mental illness, and child and young adult mortality rise with increased rurality [15].

Despite vast differences between urban and rural landscapes, several characteristics are shared among vulnerable populations throughout the US. Disparities in healthcare access and outcomes occur most frequently among inner-city and rural poor, un- and underinsured, elderly, Hispanics, and African-Americans [16]. The Affordable Care Act (ACA), which among many other things aims to mitigate insurance inequality, for example, has expanded new coverage to over 10 million Americans [17]. Despite this progress, recent studies reported mixed health outcomes among those federally insured, underscoring that access to care is a complex problem requiring a multifaceted understanding and intervention [3, 18, 19].

**Fig. 1:**
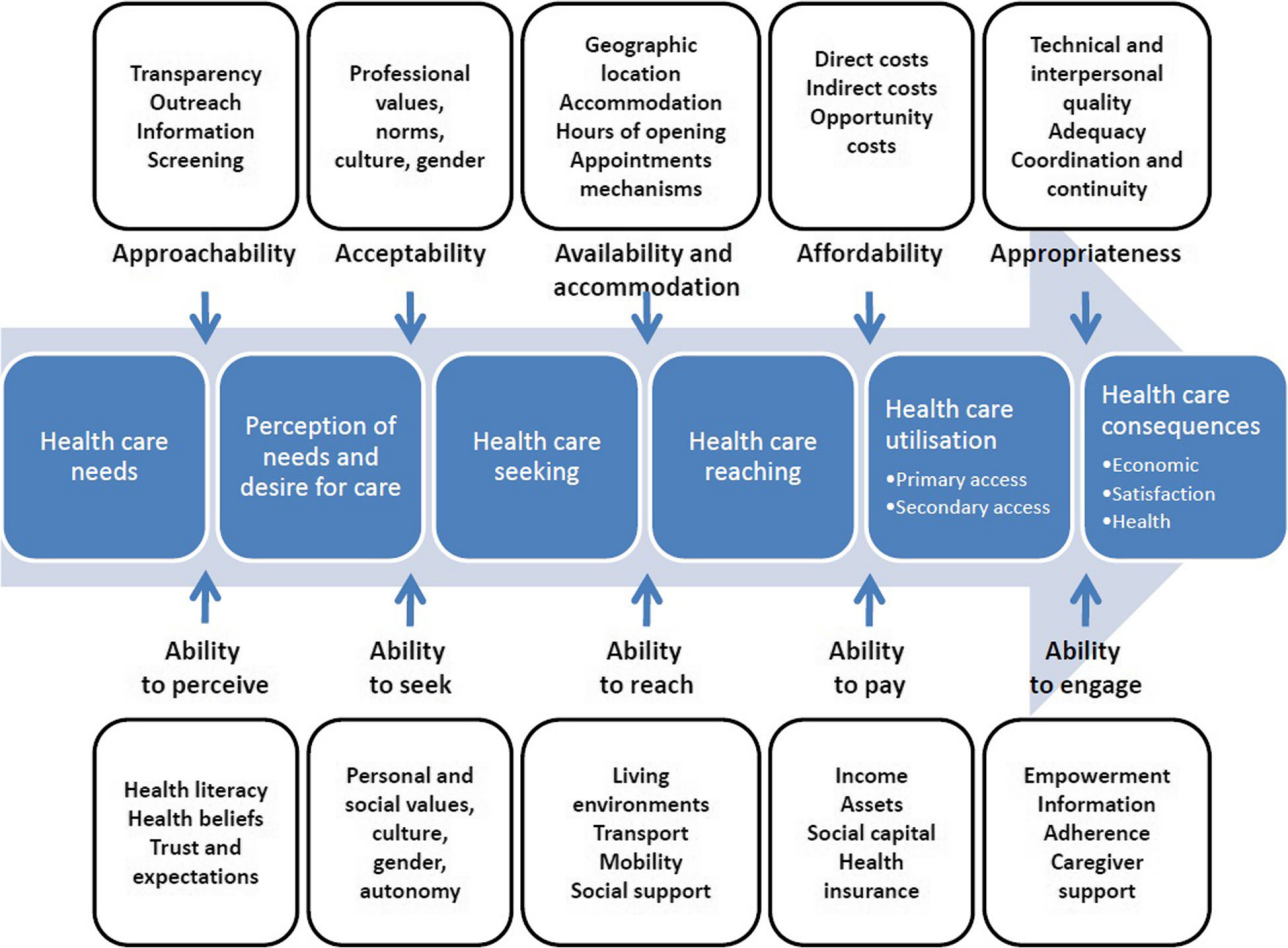
A conceptual framework of access to healthcare. Reprinted with permission from “Patient-centred access to health care: conceptualizing access at the interface of health systems and populations,” by J.-F. Levesque, M. F. Harris, & G. Russell, 2013, International Journal for Equity in Health, 12(1), 5. Copyright [2013] by Levesque et al.; licensee BioMed Central Ltd

Seminal healthcare access research originated in the late 1960s sociology literature [20] and developed over the next few decades to include variables such as organization, policy, supply and demand, population health, and economics [11, 12, 21]. Canadian authors Levesque et al. [22] more recently conceptualized access as the opportunity to identify, seek, reach, obtain, and use healthcare services. They developed a literature-driven access to healthcare framework (Fig. 1) that is based on five system “supply” dimensions (approachability, acceptability, availability and accommodation, affordability, and appropriateness) and five patient “demand” dimensions (ability to perceive, seek, reach, pay, and engage). While this framework is more holistic than earlier models, it may not completely address nuances between urban versus rural US access to specialty care.

Despite proportionately more specialized physicians in practice than in many other countries, greater disparities exist among un- and underinsured Americans versus industrialized nations with universal healthcare coverage [3]. Roughly two-thirds of practicing physicians offer specialty care services in the US [23]. Due to lower service demand, specialists and subspecialists generally cluster in more urbanized areas with larger populations to support their practice, resulting in fewer rurally located specialists and thus greater reliance on primary care providers (PCPs) [13, 15]. These observations suggest other facilitators or barriers may be unique to US specialists, PCPs, and rural versus urban areas. This systematic literature review therefore (1) thematically summarizes recent studies describing characteristics of urban and rural specialty care access in the US, (2) identifies thematic gaps in Levesque et al.’s framework [22], and (3) incorporates results into an expanded framework specific to US urban versus rural specialty care access.

## Methods

The authors collectively developed detailed study eligibility criteria prior to initiating inquiry. The Cumulative Index to Nursing and Allied Health Complete (CINAHL), Medline, PubMed, PsycInfo, and ProQuest Social Sciences databases were searched systematically using the following terms: (“health services accessibility” OR “access to care”) AND (“specialties, medical” OR “specialties, surgical” OR “specialty care”) AND (“urban area” OR “urban population” OR “urban” OR “rural population” OR “rural area” OR “rural”). Medical subject headings (MeSH) terms were used where available, and all search terms were reviewed with a health sciences research subject matter expert librarian. ‘Health services accessibility’ often is used to index articles on ‘access to healthcare’ and thus was used synonymously in our search. The Preferred Reporting Items for Systematic Reviews and Meta-Analyses (PRISMA) was used to guide results [24].

**Fig. 2:**
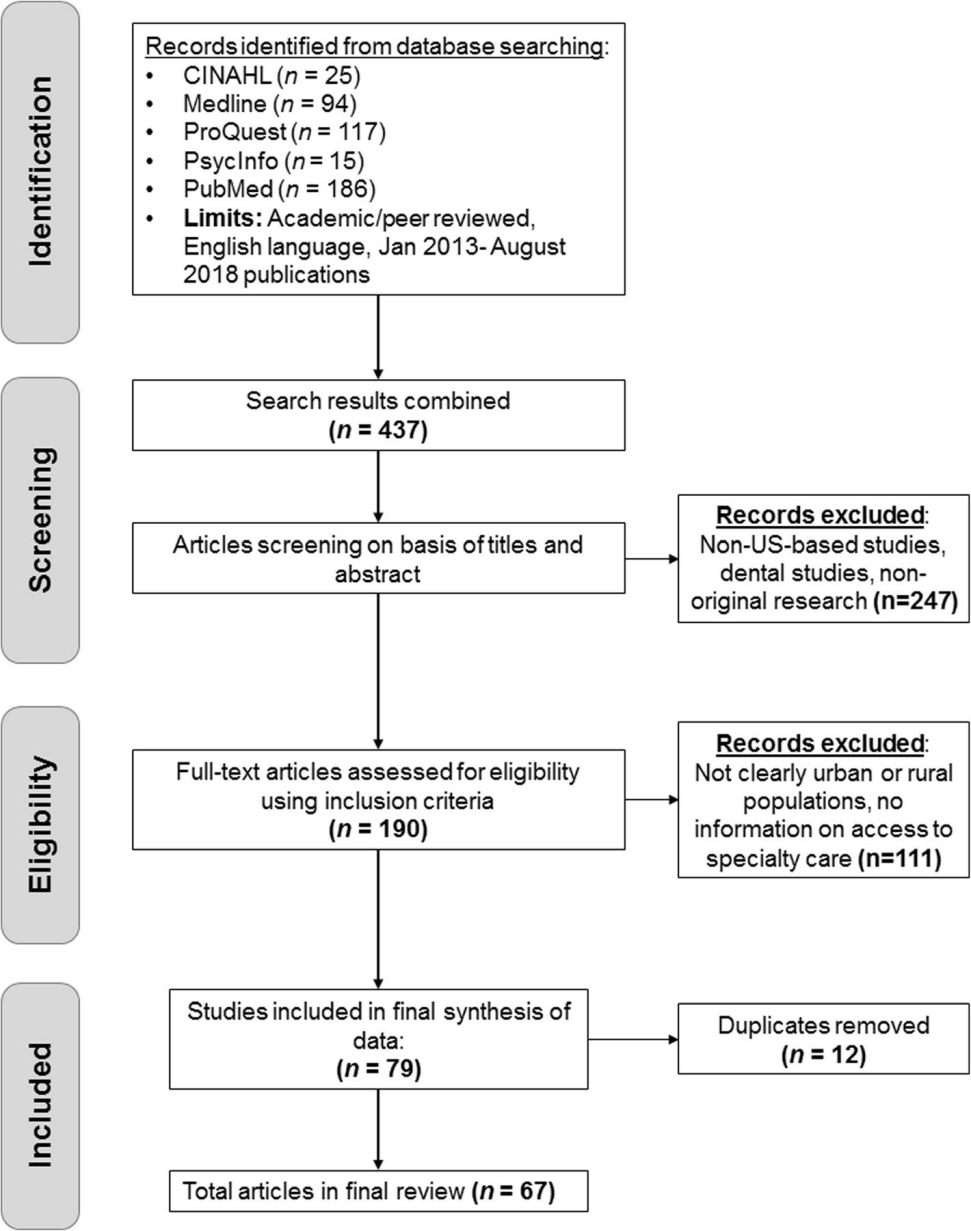
Article identification, inclusion, and selection flowchart

The search produced a total of 5709 articles (Fig. 2). Initial inclusion criteria via electronic search (peer-reviewed, English language articles published in academic journals from January 2013 to August 2018) yielded 437 results. This timeframe focused on literature following the publication of Levesque et al.’s framework [22]. All resulting titles and abstracts were reviewed by one investigator (MC) to screen for only US-based, non-dental, and original research studies (i.e., no letters to the editor), with any questionable study going to full review, resulting in 190 articles. If there were any ambiguity the paper was reviewed in full by two investigators.

The remaining full articles were reviewed independently by two authors (MC, AE) to identify descriptive findings on urban, rural, or both populations and access to specialty care. The Centers for Medicare and Medicaid Services (CMS) definitions were used: PCPs include family practitioners, general internists, preventive care, geriatricians, general pediatricians, or osteopathic physicians in general practice, whereas specialists by exclusion treat specific body systems or health problems, such as dermatologists or oncologists [25]. Access to behavioral health and other specialized services (e.g., pharmacy or rehabilitation) were included. Although emergency medical services were considered as specialized care, articles describing primary care services sought within the emergency department (e.g., treatment of upper respiratory infections) were not included. Dental literature also were excluded since delivery and insurance coverage differ from specialized medical care [26]. Preventive services performed by primary care were excluded, but screening performed by specialists were included.

The two reviewing authors discussed any discrepancies in findings to reach consensus. For each reviewed paper, data were extracted into an evidence matrix that included sample size, sample characteristics, study design, key findings, methodological limitations, population focus (urban or rural), and medical specialty foci (see summary table in Additional file 1). Additionally, these studies were independently evaluated using a quality assessment tool for evaluating primary research papers [27], with inter-rater agreement scores calculated between the two reviews and a liberal 55% cut-point was used for article inclusion. No studies were excluded due to low quality. Study findings were organized thematically with respect to Levesque et al.’s conceptual framework [22], and outliers that did not fit any of those ten dimensions were grouped using thematic analysis [28]. All results were discussed by the two lead authors (MC, AE) in terms of major findings within each existing and new dimension. Chi-square (χ^2^) testing for differences was performed on the distribution of thematic frequencies between urban, rural, and both areas and between patient, system, and emergent domains.

## Results

**Figure 3:**
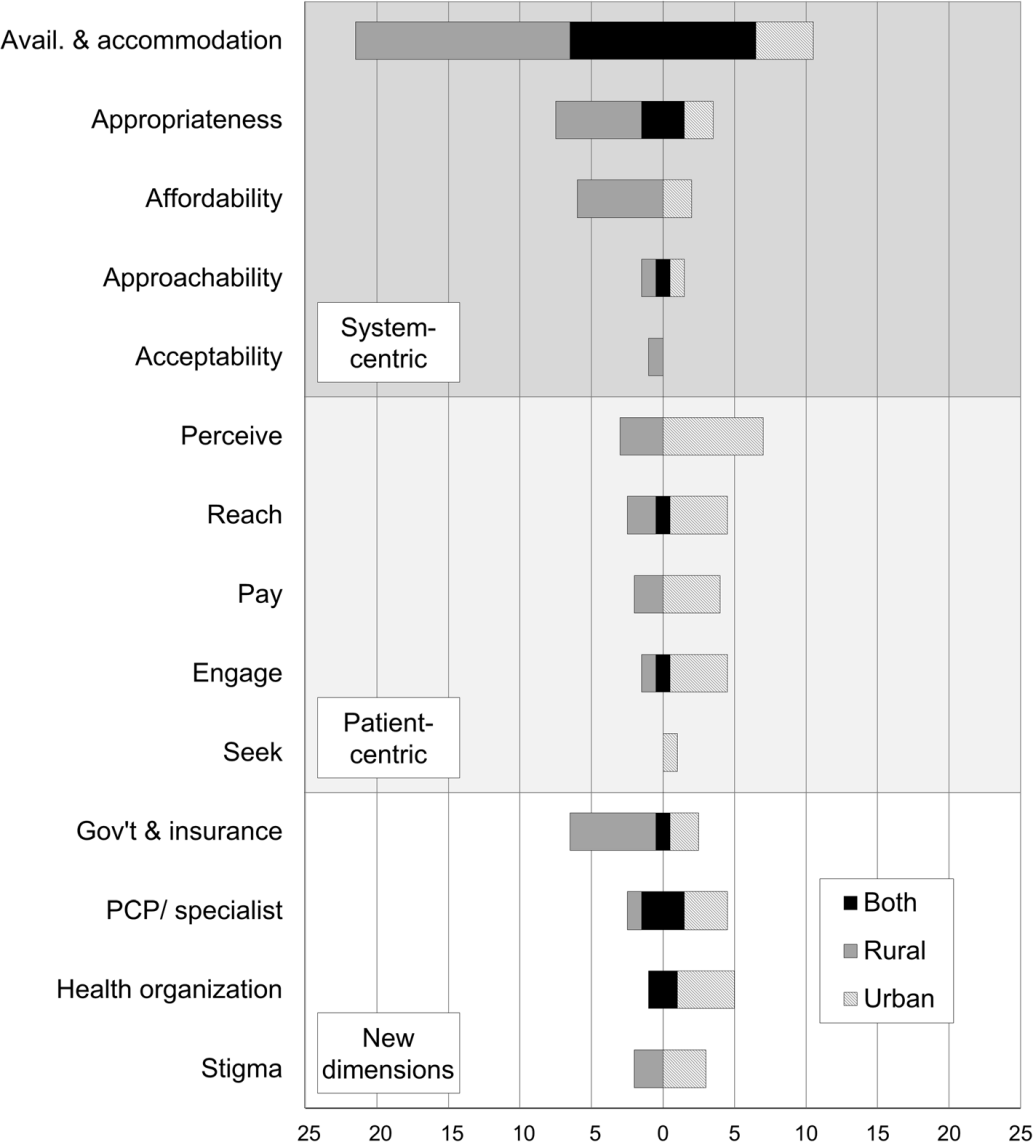
Frequency of access to specialty care dimensions, grouped by system-centric supply, patient-centric demand, and emergent themes. Avail, availability; Gov’t, government; PCP, primary care provider

The 67 reviewed articles had an average quality score of 85.7% with a low overall average inter-rater discrepancy of 8.6% (see summary table in the Additional file 2). Of these included articles, 65.7% reported results related to one or more system-focused dimensions and 28.4% reported on one or more patient-focused dimensions; 38.8% reported on urban issues, 32.8% on rural issues, and 28.4% on both (Table 1). The dimensions *availability and accommodation* (47.8%), *appropriateness* (16.4%), and *ability to perceive* (14.9%) were reported most frequently (Fig. 3). *Acceptability* (1.5%), *ability to seek* (1.5%), and *approachability* (4.5%) were discussed the least, with all dimensions discussed at least once.

**Table 1:** Healthcare access dimensions identified in the literature grouped by Levesque et al.’s [22] access to healthcare framework and emergent themes

Dimensions	% of total studies, (n)	% of urban, (n)	% of rural, (n)	% of both, (n)	References
System-centric					
Approachability	4.5 (3)	3.9 (1)	4.6 (1)	5.3 (1)	29–31
Acceptability	1.5 (1)	–	4.6 (1)	–	32
Avail. & accommodation	47.8 (32)	15.4 (4)	68.2 (15)	68.4 (13)	32,33,42–51,34,52–61,35,62,63,36–41
Affordability	11.9 (8)	7.7 (2)	27.3 (6)	–	30,32,43,64–68
Appropriateness	16.4 (11)	7.7 (2)	27.3 (6)	15.8 (3)	31,40,72,43,49,65–67,69–71
Patient-centric					
Ability to perceive	14.9 (10)	26.9 (7)	13.6 (3)	–	29,34,68,71,73–78
Ability to seek	1.5 (1)	3.9 (1)	–	–	44
Ability to reach	10.5 (7)	11.5 (3)	13.6 (3)	5.3 (1)	29,31,34,73,79–81
Ability to pay	9.0 (6)	15.4 (4)	9.1 (2)	–	29,68,71,78,80,82
Ability to engage	9.0 (6)	15.4 (4)	4.6 (1)	5.3 (1)	68,71,78,83–85
New					
Government/insurance	13.4 (9)	7.7 (2)	27.3 (6)	5.3 (1)	37,38,67,68,72,86–89
Health organization	9.0 (6)	15.4 (4)	–	10.5 (2)	33,72,90–93
Stigma	7.5 (5)	11.5 (3)	9.1 (2)	–	34,67,68,76,93
Primary care & specialist	10.5 (7)	11.5 (3)	4.6 (1)	15.8 (3)	40,67,69,72,76,94,95

Calculations are based on 67 total studies (26 urban, 22 rural, 19 of both) each reporting on at least one or more dimension. Avail, availability

The distribution of results between urban and rural regions differed both by system versus patient focus (*Χ*^2^ = 7.79, *p* = 0.0053) and by system, patient, and emergent focus (*Χ*^2^ = 8.62, *p* = 0.0134), but not between dimensions within the system (*Χ*^2^ = 1.63, *p* = 0.7950), patient (*Χ*^2^ = 1.5818, *p* = 0.8120), nor emergent foci (*Χ*^2^ = 3.96, *p* = 0.26581). These differences also are reflected in the relative lengths of each Results subsection below, summarizing key findings for each system-supply, patient-demand, and emergent dimension. Methods used in the reviewed articles included a range of quantitative (analysis of retrospective data, survey results, queried data, analytic models) and qualitative (interviews, focus groups, surveys) approaches (Table 2). Quantitative methods were used in 76.1% of included articles, with only 7.2% of those reporting patient-centric findings using qualitative or mixed-methods. Nearly 42.8% included surveys, focus groups, or interviews, whereas a few others described improvement projects, analytic models, and other methods. Again, there were no statistically significant differences in methods used by geography (*Χ*^2^ = 5.9490, *p* = 0.6529), but suggestive significance by dimension focus (*Χ*^2^ = 9.75, *p* = 0.0826), as might be intuitive.

**Table 2:** Summary of methods used in review papers stratified by study geography and dimensions

Method	Total *%* (n)	Geography *%* (n)			Dimensions *%* (n)		
		Rural	Urban	Both	System	Patient	New
Quantitative	76.1 (51)	25.4 (17)	23.9 (16)	26.9 (18)	41.0 (34)	15.7 (13)	14.5 (12)
Database	22.4 (15)	7.5 (5)	1.5 (1)	13.4 (9)	19.4 (13)	1.5 (1)	4.5 (5)
Survey	19.4 (13)	3.0 (2)	10.5 (7)	6.0 (4)	6.0 (4)	13.4 (9)	6.0 (4)
Improvement	7.5 (5)	4.5 (3)	1.5 (1)	1.5 (1)	4.5 (3)	1.5 (1)	1.5 (1)
Retrospective	19.4 (13)	11.9 (8)	7.5 (5)	3.0 (2)	13.4 (9)	6.0 (4)	3.0 (2)
Modeling	4.5 (3)	---	1.5 (1)	3.0 (2)	4.5 (3)	---	---
Other	6.0 (4)	1.5 (1)	3.0 (2)	1.5 (1)	6.0 (4)	---	---
Qualitative	19.4 (13)	10.5 (7)	9.0 (6)	---	9.6 (8)	6.0 (5)	1.2 (1)
Interviews	11.9 (8)	7.5 (5)	4.5 (3)	---	7.5 (5)	3.0 (2)	6.0 (4)
Focus groups	6.0 (4)	3.0 (2)	3.0 (2)	---	3.0 (2)	6.0 (4)	1.5 (1)
Surveys	1.5 (1)	---	1.5 (1)	---	---	---	1.5 (1)
Other	3.0 (2)	---	3.0 (2)	---	1.5 (1)	---	1.5 (1)
Mixed-methods	4.5 (3)	3.0 (2)	---	1.5 (1)	2.4 (2)	1.2 (1)	1.2 (1)

*Note:* Several studies used more than one research method and reported on multiple dimensions

### System-centric dimensions

#### Approachability

Healthcare ‘approachability’ represents the capacity of a health system to identify and provide needed services, such as transparency, information, and screening [22]. Three articles reported findings, one urban, one rural, and one on both [29–31]. Program transparency was identified as an approachability barrier for urban populations. Uninsured patients in East Baltimore, Maryland (*n* = 18) were surveyed via telephone regarding why they declined participation in a free specialty care program, with participants reporting program specific barriers, particularly no follow up and unclear participation eligibility [29]. Screening was identified as an approachability facilitator in rural populations. One case report described a Veterans Health Administration (VHA) ophthalmological asynchronous store-and-forward eye screening program in a subset of rural Georgia primary care clinics to improve access. Of those veterans screened (*n* = 1443), 10.6% were homeless, almost 5% had not received an eye screening in the preceding 10 years, 10% experienced delays greater than 30 days, and 38.8% were referred for further evaluation of underlying disease [30]. In another VHA study examining the use of e-anesthesia consults (*n* = 7988) across several New England medical centers (including urban and rural), low-risk surgical patients were screened for appropriateness for e-consults, which reduced patient travel and time to surgery and improved anesthesiologist efficiency [31].

#### Acceptability

‘Acceptability’ relates to the influence that provider characteristics, such as culture and gender, have on a patient’s acceptance of services [22]. One rural article interviewed patient perceptions of local clinicians. Adult survivors of childhood cancer in rural Utah (*n* = 17) suggested that health problems faced by cancer survivors require a level of specialization not available to them locally, with many traveling outside their rural community given limited availability of local specialists [32].

#### Availability and accommodation

‘Availability and accommodation’ relates to the timely attainment, geographic location, hours of operation, and capacity of services offered [22]. Thirty-two papers reported geographic barriers to urban care (4), rural care (15), or both (13), with the predominant specialties described being mental health and substance abuse treatment. One large study examined the geographic availability of urban and rural mental health facilities that accept Medicaid (*n* = 9696) and found that for every one standard deviation increase in the percentage of rural residents, the likelihood that the corresponding area lacked these services nearly doubled [33]. Rural counties in Wisconsin were significantly less likely than urban counties to have substance abuse treatment facilities (*r* = − 0.42, *p* = 0.00) [34], and similarly rural areas in Washington state had significantly fewer opioid agonist therapy facilities than urban (IRR: 0.03; *p* = 0.002) regardless of whether its status was an American Indian (AI) reservation (IRR: − 0.66; *p* = 0.79) [35]. Lastly, the geographic supply and distribution of behavioral health professionals in Nebraska were examined, and while rural areas had roughly one-third as many specialists per capita compared to urban areas, only very rural frontier areas (≤6 persons/square mile) met the federal mental health processional shortage area’s definition of underserved (< 3.3 providers per 100,000), here with 2.2 providers per 100,000 residents [36].

Geographic service barriers also were reported in three maternal and pediatric articles. Only 18% of the high maternal risk, rural dwelling females studied (*n* = 16,363; Colorado, Iowa, Kentucky, New York, North Carolina, Oregon, Vermont, Washington, and Wisconsin) resided within 30 miles of an advanced neonatal care facility, and only 40–43% delivered in a setting that offered this specialized care [37]. Among 4,941,387 births studied, loss of hospital-based obstetric services in remote rural areas was associated with increases in out-of-hospital births (0.70 percentage points (95% CI, 0.30–1.10)), in-hospital births without an obstetric unit (3.09 percentage points (95% CI, 2.66–3.46)), and preterm births (0.67 percentage points (95% CI, 0.02–1.33)) [38]. Another study examined women seeking care for gynecologic malignancies at an urban medical center (*n* = 152); while the majority of patients completed the recommended therapy (87%), those who died prior to completion (5.3%) lived the furthest from care, whereas patients who did not complete treatment (7.3%) lived the closest [39], underscoring that accessibility and availability are not as simple as proximity. In a study examining the management of children with special healthcare needs, 81% of urban and rural Montana PCPs (*n* = 91) reported a lack of appropriate local specialists as one of the top barriers to care, with long wait times of travel times identified by 63 and 60%, respectively (no significant differences between urban/rural) [40].

Three studies described geographical barriers to care in veteran populations. Over 83% of rural dwelling veterans (2312 rural; *n* = 23,639) with HIV were found to reside more than 60 min from infectious disease specialists, with care utilization rates roughly 17% lower for those living 90 versus 15 min from care (*p* < 0.01) [41]. Similarly, 75.4% of rural Veterans eligible for purchased care under the US Choice Act resided in mental healthcare shortage areas, including 73.3% in areas without a practicing psychiatrist [42]. A large national study of veterans with multiple sclerosis (*n* = 14,723) found that while 65.5% received specialty care related to their diagnosis, those who experienced military-related injury or illness, lived in urban areas, or had greater medical comorbidity were more likely to have received care, whereas older veterans and those traveling greater distances were less likely [43].

Traveling for care also was a central theme in six other articles. Semi-structured interviews with rural adult childhood cancer survivors sampled from the Utah Cancer Registry (*n* = 17) found that many traveled to larger cities for care, although few described this as burdensome rather than just a consequence of rural residence [32], again highlighting that access is not as simple as proximity alone. Compared to rural patients, longer travel to radiation facilities for urban patients with prostate cancer was associated with a lower likelihood of choosing external beam radiation therapy in a New Hampshire study (*n* = 4731) [44]. In contrast, rural Virginia dwelling patients with cervical cancer living further from a treatment center were not more likely to be diagnosed at more advanced stages, experience longer times to complete treatments, nor experience poorer outcomes [45]. In a study of southern US HIV treatment facilities (*n* = 228), rural counties with highest disease prevalence rates had median travel times exceeding 60 min, more than seven times than in super-urban areas [46]. In semi-structured telephone interviews with rural practicing pediatricians (*n* = 17), respondents resided an average of 2.4 h from the nearest tertiary care center and 71% had no local pediatric subspecialists so used adult specialists instead [47]. Finally, a computer model that examined accessibility and availability of specialty care in several US cities using data from the Cystic Fibrosis Foundation found that while urban areas have greater appointment availability, access delays still occur due to congestion and travel time [48].

Six studies described specialty availability in terms of local population race and ethnicity. Researchers in Los Angeles, California examined clinics in geographical ethnic hot spots (*n* = 402) for the availability of integrated mental health and substance abuse treatment programs (*n* = 104) and found that only 20% of poor, urban, Latino community clinics offered integrated care (OR: 0.28, 95%; CI: 0.09–0.85) [49]. Hispanics and non-Hispanic Blacks experienced greater travel times due to geographic disparities of urban HIV treatment facilities in southern US counties (*n* = 228 locations) [46]. Rural dwellers, minorities, and socioeconomically disadvantaged individuals disproportionately reside in areas without emergency general surgery hospitals (*n* = 2811 US hospitals) [50]. Rural dwelling females across nine states (*n* = 37; Colorado, Iowa, Kentucky, New York, North Carolina, Oregon, Vermont, Washington, and Wisconsin) with high maternal obstetric risk had the greatest odds of giving birth in a hospital without neonatal intensive care services were less than 20 years old (OR: 0.87, 95%; CI: 0.77–0.98), Medicaid beneficiaries (OR: 0.81, 95%; CI: 0.75–0.89), Black women with preterm delivery (OR: 0.60, 95%; CI: 0.50–0.71), or self-pay or uninsured individuals (OR: 0.44, 95%; CI: 0.32–0.61) [37]. In a study that examined the relationship between race, urban and rural geography, and quality-of-care in patients approaching end-stage renal disease (*n* = 404,622), rural areas examined had fewer healthcare resources and only one-third as many nephrologists; regardless of race, access to specialty care was worse in large urban and rural counties, and for all measures of care Black patients were less likely than Caucasians to have received care regardless of urbanicity (*p* < 0.001) [51]. In an urban study that examined geographic disparities in mental healthcare (*n* = 12,395), geographic ethnic density was a statistically significant negative predictor of specialty service availability [52].

Six studies tested interventions that sought to increase the geographic availability of specialty care. Acute stroke access program implementation in one rural western North Carolina emergency department was found to increase care and reduce numerous measurable time-based metrics, such as time to neurological imaging, administration of intravenous thrombolytics, and transfer to a more specialized facility [53]. Implementation of rural telehealth programs also were shown to increase access to specialty care in mental health evaluation in a critical access emergency department [54], medical abortion services in Iowa [55], nephrology care in upstate New York veterans [56], and rheumatology care in New England [57]. Similarly, an urban teledermatology program in Philadelphia provided 11 underserved clinics with access to dermatology care [58].

Six papers addressed specialist rural outreach. In four studies examining specialist (otolaryngology, oncology, orthopedic, and cardiologist) outreach in rural Iowa, roughly 45–46% of specialists engaged in outreach services and traveled 17,000 (otolaryngologists) to 45,000 miles (cardiologists) per month, reducing patient driving burden (up to 19.2 miles per patient monthly in orthopedics) and increasing care to over 1 million patients (otolaryngology and cardiology) [59–62]. In a similar study in Iowa, oncologists were found to be distributed primarily in urban or large rural areas [63]. Of Iowa residents newly diagnosed with invasive cancer (*n* = 113,885; 2004–2010), rural dwellers drove three times longer than urban for care, but in areas offering oncologist outreach, 24.2% underwent therapy near their home versus only 10.3% if no specialist was available [63].

#### Affordability

‘Affordability’ refers to direct, indirect, and opportunity costs associated with care delivery [22]. Service reimbursement rates and practice costs were reported barriers to specialty care delivery in 8 articles (2 urban, 6 rural). Interviews with obstetricians, certified nurse midwives, and maternal and infant health leaders (*n* = 46) in rural Georgia found that Medicaid reimbursement rates were too low and the cost of malpractice was too high to continue providing obstetric care [64]. One study that examined urban specialty practices throughout Cook County, Illinois (*n* = 273; allergy/pulmonary, dermatology, endocrinology, neurology, orthopedics, otolaryngology, and psychiatry) found that clinicians were less likely to deny appointments to publicly insured children in areas with greater neighborhood poverty (OR: 0.95, 95%; CI: 0.93–0.98) and specialist density (OR: 0.74, 95%; CI: 0.57–0.98) [65]. Semi-structured interviews with rural dwelling adult childhood cancer survivors (*n* = 17) found that rural residence sometimes created financial difficulties associated with emergency travel and care [32]. Focus groups with urban dwelling Mexican immigrants in North Carolina (*n* = 81) found that care received from emergency departments provided less favorable experiences than from community health centers due to higher care costs [66].

Four studies reported the reduced travel costs and improved access due to telemedicine. Interviews with rural Iowa and Illinois veterans with HIV (*n* = 13) found that specialty care telehealth access resulted in reduced median yearly travel time (150 min), time away from work, and travel costs [67]. In other VHA telemedicine studies, a remote eye screening program for rural dwelling veterans reduced the volume of necessary face-to-face visits and overall healthcare costs, saving approximately $150 per visit and $52 per patient in travel [30], and a urology program (*n* = 97) reported a savings of approximately $126 in opportunity costs per appointment [68]. Similarly, in a three-year rural New England study, rheumatology patients (*n* = 176) lived on average 99 miles from their rheumatologist and 22 miles from their primary care provider, with implementation of a telerheumatology program saving the health system almost $27,000 in consulting specialist travel (roughly $67 per visit) [57].

#### Appropriateness

‘Appropriateness’ is defined as the fit between an individual’s needs and services, as well as the quality of these services [22]. Eleven articles reported findings in this dimension (2 urban, 6 rural, 3 of both). A study of recent stroke survivors and health system stakeholders (*n* = 52) from rural South Carolina described the need for improved communication and relationships among healthcare providers, and between healthcare providers, patients, and their family [69]. In a study examining children with special healthcare needs in urban and rural Montana, primary care providers (*n* = 91) reported that approximately 17% of their practice time was spent coordinating care with specialists, with ease of communication and care quality important in choosing specialists for referrals [40]. In 11 rural and urban Nebraska pediatric clinics, 96% of examined PCPs (*n* = 27) were satisfied with the overall care quality of integrated behavioral health services and 93% reported improved care continuity [70].

The quality and coordination of telehealth care delivery was another important theme reported in four articles. Rural dwelling Iowa and Illinois veterans with HIV studied (*n* = 13) who sought telehealth infectious disease specialty care reported high program satisfaction overall (78%), increased trust in providers and care continuity, and appreciation for the opportunity to discuss treatment advances with specialists, as well as identifying occasional care coordination difficulties that were acceptable tradeoffs for the added convenience [67]. In a VHA study that examined the use of e-anesthesia consults for low-risk surgery in urban and rural New England patients (*n* = 7988), anesthesiologists reported no adverse events occurred attributed to the e-consults, possible issues were identified weeks before a procedure rather than days, fewer surgeries were cancelled, and patient-centeredness improved [31]. In a feasibility study of a rural VHA telemedicine urology program (*n* = 97 patients), overall satisfaction scores (94–100%) were high across numerous metrics, such as a 97% program recommendation rating, with only one required emergency department evaluation for hydronephrosis within 1 month of evaluation [68]. However, in a telerheumatology program in rural Vermont and New Hampshire (*n* = 176 patients, 244 visits), while the majority of patients were managed appropriately (81%), only 53% of surveyed patients agreed or strongly agreed they would like to be seen via telehealth again, with lower scores attributed to issues establishing follow-up care [57].

Four studies reported barriers to care continuity. Logistical barriers to post-traumatic stress disorder care in urban dwelling veterans in Portland, Oregon (*n* = 63) contributed to poor engagement in patient-provider relationships, treatment non-receipt, and program drop-out [71]. Pediatricians (*n* = 17) across 17 rural states identified that the ability to share medical records and communicate with subspecialists was important for care continuity [47], and obstetric clinicians and health leaders (*n* = 46) in rural Georgia underscored that care continuity also was a challenge in their practices [64]. In terms of improvement strategies, an urban New York needs assessment identified that well-defined roles and effective communication were essential to collaboratively managing complex psychiatric care needs [72].

### Patient-centric dimensions

#### Ability to perceive

‘Ability to perceive’ care needs relates to issues that influence approachability, such as health beliefs, literacy, and expectations [22]. Ten articles reported findings in these areas (7 urban, 3 rural). Despite initial apprehensions, many women interviewed in rural Iowa (*n* = 25 women; *n* = 15 staff) reported an overall positive experience with an abortion telemedicine program that allowed for more timely procedures [55]. Another study that examined health beliefs of medically indigent patients in a free otolaryngology Chinatown clinic in Philadelphia found little consistency between which specialty care services patients versus clinicians believed were needed [73]. In a study of rural dwelling adult female veterans (*n* = 35; North Carolina, Colorado, Georgia, Hawaii, California, Washington, and Texas), many reported an unawareness of available VHA benefits [74]. Recent stroke survivors and health system stakeholders in a low income, rural area in South Carolina reported patient-centered barriers while seeking acute stroke care (*n* = 52) [69], including a lack of trust in the healthcare system and providers, misinformation about insurance utilization in the emergency department, and a belief their needs were inconsistently met; additionally, healthcare providers reported low health literacy in stroke symptoms recognition and when to seek care [69]. Focus groups with urban dwelling Mexican immigrants in North Carolina (*n* = 81) found that while there was some confusion about health insurance and coverage, many believed insurance is necessary to avoid high medical bills, have better access to medical care, and should be prioritized most for children [66].

Five studies also described the importance of parents’ perceptions of specialty care for children. Parents whose children were referred to an integrated behavioral health program in urban Baltimore City, Maryland (*n* = 55) reported high-levels of intangible barriers, such as worry a child may require medication (13%) or belief specialty care was not warranted (12–15%), resulting in a decreased odds of engaging in care (OR: 0.20, 0.06–0.83; *p* = 0.03) [75]. Focus groups with Latino and African-American parents in Dallas, Texas (*n* = 267) found that while almost 66% of children had specialized health needs, over half were unmet due to problems obtaining a referral, no insurance, Medicaid eligibility unfamiliarity, or inability to receive after hours assistance [76]. Focus groups with parents of Iraqi refugee children (*n* = 24) in urban Dallas, Texas suggested a poor understanding of the US healthcare system, difficulty navigating referrals, frustration with long appointment delays, few local clinicians accepting Medicaid, or the inability to distinguish differences between generalists and specialists [77]; results of semi-structured interviews with care staff (*n* = 8) in the same study also include long waits for specialty appointments, parents uninformed of clinic location changes, and visits that felt rushed with inadequate time to ask questions while using interpreters. Results that aimed to understand why eligible patients (*n* = 18) were not participating in a free specialty care program in East Baltimore, Maryland included referral or eligibility misunderstanding, forgetting follow up, beliefs that services were not needed, and preferentially seeking care elsewhere [29]. Finally, a substantial portion of respondents in a cross-sectional study of children (*n* = 756) in a low-income, Midwestern US city reported uncertainly about insurance coverage for rehabilitative services, with those being covered 1.7 times more likely to participate in care [78].

#### Ability to seek

‘Ability to seek’ encompasses one’s culture and healthcare values, capacity to autonomously seek care, and patient-centered appropriateness of care [22]. Only one urban article was identified in this dimension, a large secondary analysis of survey data (*n* = 12,395) describing cities with cultural disparities in mental health care utilization, most notably Black-White disparities in Richmond, Virginia and Columbus, Georgia; Latino-White disparities in Fresno and Los Angeles, California and Houston, Texas; and Asian-White disparities in Fresno and Riverside, California and Houston, Texas [52].

#### Ability to reach

‘Ability to reach’ includes issues such as mobility and transportation and is related to an individual’s physical ability and social support reaching these services [22]. Seven articles identified findings pertinent to this dimension (4 urban, 2 rural, 1 of both). In a study of White and American Indian/Alaska Native children who completed inpatient rehabilitation (*n* = 1257), roughly 85% resided in rural, remote, or reservation areas that lacked specialized rehabilitation services and the physical environmental to support mobility assistive devices, such as wheelchairs and walkers [79]. One’s living environment, stability, and support also may contribute to specialty care accessibility; a retrospective study of formerly homeless individuals in urban Portland, Oregon (*n* = 98) had significant medical and psychosocial challenges prior to moving into supportive housing, including addiction (51%), incarceration (41%), sexual abuse (20%), and higher than average healthcare costs (3.5 times) [80]. However, move-in participants subsequently experienced significant (*p* < 0.05) reductions in health costs, improved mental health service utilization, reduced emergency department visits, and overall improved subjective health and happiness [80].

Most articles relevant to this thematic dimension also included travel or transportation issues. Rural adult female veterans (*n* = 35; North Carolina, Colorado, Hawaii, Georgia, California, Washington, and Texas) reported that the extended travel required to utilize VHA services contributed to work and childcare conflicts, and while over half had favorable views of telehealth, local in-person care was preferred with the top requests being for dental (*n* = 26), mental health (*n* = 23), contraception/family planning (*n* = 22), and domestic/interpersonal violence services (*n* = 19) [74]. Another VHA-based study in urban Portland, Oregon (*n* = 63) reported multiple barriers to veterans engaging in PTSD psychotherapy services, such as conflicting personal commitments (i.e., work, school, family responsibilities, or lack of child care), limited financial travel resources, medical problems that interfered with long distance travel, deployment anticipation, or legal issues (e.g., loss of driver’s license or incarceration) [71]. In another VHA study, anesthesia e-consults prior to low risk surgery that reduced the need for unnecessary travel and multiple appointments were favorable to patients [31]. In urban East Baltimore, Maryland, some eligible participants (*n* = 18) were not utilizing free specialty care services because they were too sick to attend appointments or had transportation or mobility issues [29]; homeless patients (*n* = 200) in Birmingham, Alabama similarly did not access specialty (45%) and mental health (43%) care primarily due to a lack transportation [81]. Iowa females seeking medical abortion services (*n* = 25) and their clinical stakeholders (*n* = 15) reported factors for choosing telemedicine included close proximity, reduced time off from work or school, fewer travel costs, inability to drive (e.g., no license), and not having to explain reasons for travel [55].

#### Ability to pay

‘Ability to pay’ reflects an individual’s economic capacity and willingness to participate in and pay for care [22]. Note that affordability reflects a provider’s direct, indirect, and opportunity costs of offering care, whereas ability to pay reflects an individual’s direct, indirect, and opportunity costs with affording care services. Six articles reported findings in this dimension (4 urban, 2 rural), including multifactorial economic factors beyond inability to pay. Some eligible patients (*n* = 18) in East Baltimore, Maryland not utilizing specialty care services were unable to afford enrollment fees or experienced work conflicts [29]. Recent stroke survivors and stakeholders (*n* = 52) from rural South Carolina similarly reported inability to risk missing work, limited insurance or burdensome out-of-pocket expenses, worry about how bills will be paid, and inability to pay for medications [69]. Eighty-three Birmingham, Alabama urban homeless individuals (*n* = 200) reported barriers to specialty care, including inability to pay (64%) and safety-net insurance not being accepted (46%), while 46% of 77 cited barriers to mental health care as inability to pay [81].

Beyond financial ability, values placed on individual care services and insurance processes may limit willingness to pay. On average, surveyed patients across rural Kentucky (*n* = 796; 10 counties) indicated a willingness to pay for cancer care services and forgo spending on dialysis or physical therapy, services they may not prioritize or require (results were not statistically significant) [82]. Similarly, beyond the cost of obtaining health insurance, many interviewed working class Mexican immigrants in urban North Carolina (*n* = 81) feared illness and the inability to pay for care [66]. Assisting staff and parents of Iraqi child refugees in urban Dallas, Texas (*n* = 24) reported that while 67% had Medicaid, this insurance itself created difficulty finding participating psychologists, along with burdensome renewal processes leading to coverage lapses [77].

#### Ability to engage

‘Ability to engage’ refers to an individual’s ability and motivation to participate in treatment decisions and care [22]. Six articles (4 urban, 1 rural, 1 of both) reported findings in this dimension, with information being the predominant factor. Recent stroke survivors, particularly those who are elderly, may not understand provider care instructions [69]. Mexican immigrants in urban North Carolina (*n* = 81) reported communication as one barrier to seeking health insurance; often Spanish speaking personnel were limited in availability and poorly equipped to answer specific questions [66].

Caregiver information and empowerment themes also were identified in the literature. Language was a barrier to healthcare engagement for Iraqi refugee families in Dallas, Texas (*n* = 24), with inadequate interpretation services and non-Arabic health education materials, whereas they favorably viewed assistance they received (transportation, interpretation services, and specialist access) [77]. Several family-centric barriers were reported as reasons for failed follow up in high-risk ophthalmological pediatric patients in urban Philadelphia, Pennsylvania (*n* = 93), including lack of awareness of follow up necessity (13%), assumption there would be a reminder (5%), scheduling conflicts (4%), concerns about insurance (2%), and difficulty finalizing referrals (2%) [83].

In terms of health literacy, self-management, and self-efficacy, patients in an urban Alabama glaucoma clinic with higher education had statistically lower satisfaction scores with appointment accessibility and convenience [84]. Fewer Missouri and Alabama rural dwelling residents after tornado disasters (*n* = 676) accessed online mental health interventional materials compared to urban and suburban (*R* [2] = 0.002) [85].

### Emergent dimensions

#### Government and insurance policy

Nine articles (2 urban, 6 rural, 1 of both) described how government or insurance policies affect care access and delivery. Two of these described the influence government funded medical training programs and financial incentives may have on the availability and distribution of specialists. Graduate medical education funding was redistributed in 2005 in an effort to increase rural training (*n* = 304 affected hospitals), but CMS data from 1998 to 2009 indicate that while 83 new primary care training positions were created, almost 495 primary care programs were converted to specialist training [86]. Twenty-four-percent of Georgia obstetrician/gynecologist residents (*n* = 95) and 54% of certified nurse midwife students (*n* = 28) expressed interest in practicing in rural Georgia (*p* < .001), with 89 and 96% respectively indicating greater likelihood of practicing in rural Georgia if offered financial incentives [87].

Five articles described the influence that reimbursement and insurance policies have on access to quality specialty care. A study in rural Iowa suggested the ACA may reduce reimbursement for visiting otolaryngologists and thus reduce access to care in vulnerable populations [61], although a similar rural Iowa study that found medical oncology access increased significantly after the Medicare Modernization Act (2005) [62]. Interviews with rural Georgia obstetric clinicians (*n* = 46) found that some patients with high-risk pregnancies do not receive care until well into their second trimester because of a long Medicaid application process [64]. Global reimbursement policies also can create financial advantages for clinicians to keep higher-risk patients under their care rather than refer them, even if the latter is in the best interest of their patient [64].

In terms of legislation as a barrier, interviews with VHA staff (*n* = 43; rural West, South, and Midwest US) about the effects of the Veterans Choice Act to allow veterans to seek care outside the VHA suggested three reasons it resulted in even longer access delays: 1) the policy was implemented too quickly without adequate preparation; 2) external care relied upon already overburdened community providers; and 3) communication and scheduling barriers existed outside the VHA [88]. Mexican immigrants (*n* = 81) in an urban North Carolina study suggested that by providing proof of insurance one would not have to provide a Social Security number, a common barrier to seeking healthcare [66]. Forty-seven-percent of Georgia obstetrician/gynecologist residents (*n* = 95) and 32% of certified nurse midwife students (*n* = 28) indicated they were less likely to practice in Georgia because of unfavorable political and social environments that restricted reproductive rights [87]. Complicated licensing and outdated billing structures were reported as barriers to creating integrated primary and behavioral healthcare clinics in urban New York [72].

Finally, governmental influence on clinician practice and satisfaction was found to influence specialist access. Among actively practicing physicians across Pennsylvania (*n* = 17,444), 12% reported career dissatisfaction and 18% plan to leave patient care over the next 6 years, with higher odds in each for rural versus urban practitioners (*p* < 0.1) [89]; reported causes for career dissatisfaction included lack of leisure time, governmental regulations, bureaucracy, administration, paperwork, and fear of litigation [89]. Obstetricians and maternal-fetal medicine specialists in a rural Georgia study similarly reported fear of potential malpractice leading to defensive medicine and increased patent-provider mistrust [64].

#### Health organization and operations influence

Six articles (4 urban, 2 of both) reported findings related to health organizational and operational influence as creating or removing barriers to specialty access. Improvement of schedule, greater use of advanced practice clinicians in an urban North Bronx, New York City practice improved monthly patient volume from 284 to 374 (mean), reduced wait times for new appointment wait times from 11.0 to 1.7 weeks (*p* < 0.001), and reduced follow up wait times from 8.2 to 2.9 weeks [90]. With respect to difficulty hiring specialists due to lower salaries, a study of 18 urban safety-net health systems in 10 states (California, Washington, Massachusetts, Georgia, Texas, Michigan, New York, and Minnesota) improved access by offering telehealth and electronic consults, co-locating PCPs with specialists, and discharging specialty care patients back to their PCP when clinically indicated [91].

A study contrasting urban and rural obstetric and gynecological practices (*n* = 73) in five Pacific Northwest states found that recruitment patterns impacted specialist availability, with urban clinicians seeking partners with more specialized skills and rural clinicians being more likely to leave their practice due to poor specialized care access [92]. Some organizations may choose to limit patient access based on insurance, such as one-third of the counties in a large urban and rural study of mental health facilities (*n* = 9696) that did not accept Medicaid [33] or urban non-profit general care hospitals studied in Philadelphia (*n* = 15) that should offer more affordable and publically funded substance abuse and mental health services [93]. Organizational culture and leadership also were reported as major mediators in an urban New York needs assessment of integrated primary and behavioral healthcare [72].

#### Stigma

Five studies (3 urban, 2 rural) reported patient perceived stigma or clinician discrimination about a medical condition or service. Due to internalized or anticipated stigma, two women in rural Iowa (*n* = 25; *n* = 15 clinical staff) opted to receive telemedicine abortion care and noted this allowed for easier discussions with their clinician [55]. Fifteen-percent of parents of children who required mental healthcare in urban Baltimore, Maryland (*n* = 55) reported their family and friends would not support their decision to pursue recommended treatment [75]. Stigma associated with seeking care was identified as a barrier to substance abuse treatment in an urban Philadelphia needs assessment [93] and VHA PTSD treatment in urban Portland, Oregon (*n* = 63) [71]. Interviewed obstetric care providers in rural Georgia (*n* = 46) found some perceived that lower socioeconomic populations were less likely to adhere to risk-reduction suggestions and more likely to arrive late or miss appointments [64]. Urban dwelling Mexican immigrants in North Carolina (*n* = 81) who sought emergency or urgent care reported discriminatory treatment contributing to negative overall care experiences [66].

#### Primary care and specialist influence

Seven studies (3 urban, 1 rural, 3 of both) described the influence PCPs or specialists have on a patient’s utilization of specialty services. In a study examining the management of children with special healthcare needs, opinions of urban and rural Montana PCPs (*n* = 91) were found to have an important role in specialist utilization, particularly regarding specialty subtypes, quality of care provided, communication ease, care coordination, and geographic proximity [40]. In a similar study, PCP (*n* = 27) opinion and satisfaction in 11 rural and urban Nebraska pediatric clinics were viewed as important for integrated behavioral health service utilization [70], while referrals to a pediatric behavioral health program in urban Baltimore, Maryland were at the discretion of the child’s PCP [75]. Primary care referrals also were necessary for patients to participate in telepharmacy VHA services (*n* = 711,348) with urban dwelling patients more likely to participate (24.9% vs. 19.7%; OR = 1.35) [94]. In a study of an integrated primary and behavioral care model in urban New York, some psychiatrists were more insular and provided consultation only, with others taking a team approach to care [72]. Patients who received a gastroenterology referral (*n* = 266) in an urban San Francisco, California but were not seen because, while most referrals (62%) were PCP generated, 32% of these were deemed as not requiring a specialty referral, 31% were incorrectly referred, and 6% should have been referred to another specialty [95].

Specialists’ perceptions were a final reported access barrier. A study of rural Georgia obstetricians, certified nurse midwives, and maternal health leaders (*n* = 46) found that older physicians were reluctant to work with nurse practitioners or physician assistants, resulting in concerns about care quality during labor and delivery, patient role confusion, and less collaboration; however, nurse midwives felt they could be more effectively utilized for routine first-line care, freeing up obstetricians for higher risk cases [64].

## Discussion

Barriers to specialty care access are pervasive and multi-factorial, with consequences on timely care, outcomes, and equity. This systematic review summarized recent access to US urban versus rural specialty care literature and thematically organized results within an existing conceptual framework in other care access contexts [96, 97]. Implications of results are three-fold. First, results largely support the framework proposed by Levesque et al. [22], as well as identify important gaps unique to US urban and rural access to specialty care. Of the 67 studies we included, 26 described barriers in urban populations, 22 rural, and 19 in both. Despite notable differences between urban and rural services, structure, and populations, results suggest that individuals residing in urban and rural areas experience both similar and different challenges to healthcare access. Although the *acceptability, ability to seek, and health organization influence* dimensions were reported in either only the urban or rural literature, all other dimensions were described in both geographic areas. We believe this may be of particular importance to public health officials and policy makers when planning more generalizable initiatives targeting large scale access to care improvement.

Second, these results further underscore that healthcare access is not a simple concept. For example, residing in a rural geographic location was not related clearly to diagnostic delays, greater morbidity, nor mortality in the literature. Rural patients that traveled further for cervical cancer treatment were no more likely to experience delayed diagnosis until an advanced stage, reduced overall survival, greater progression risk, nor longer treatment [45]. Primary care clinicians also may function as specialists due to necessity in underserved settings, such as for rural dwelling children with special health needs [40] and rural dwelling veterans with HIV [41]. However, primary care clinicians are already resource constrained with long appointment wait times reported through the US [98], and they unlikely possess the training necessary to manage all specialty care needs locally [1, 8]. Due to long travel distances, several remote primary care facilities offer integrated behavioral healthcare [70] or telemedicine services such as ophthalmologic eye screening [30] or telerheumatology [57]. Unfortunately, further insurance and policy changes are warranted as regulatory implications and reimbursement limitations still persist throughout much of the US [99, 100].

Third, several new themes that impact access were identified that may be important to integrate into conceptual frameworks for improving our understanding of care access. Government and insurance policies may facilitate or restrict access through training resource allocation [86], financial incentives [87], insurance policy [61, 64], reimbursement [64, 72], legislation [66, 72, 87, 88], malpractice [64, 89], and increased government oversight [89]. Health organization and operations influence may include organizational culture or leadership [72], process and performance initiatives [90, 91], specialist recruitment strategies [91, 92], strategic geographic location [33], insurance acceptance [33], and decisions regarding offered specialty services [93]. Patient perceived stigma with a medical condition or service [55, 66, 71, 75, 93] and clinician directed patient discrimination [64] also were identified barriers. Finally, primary care and specialty clinicians themselves were found to influence access [40]. Opinion and satisfaction with care coordination [40, 70], required PCP generated specialty care referrals [94, 95], and engagement with integrated specialty care delivery programs [72] were identified as primary care themes affecting access, while one study described how specialists’ perceptions also may create reluctance among some physicians to collaborate with nurse practitioners, physician assistants, or other physician specialists [64].

**Fig. 4:**
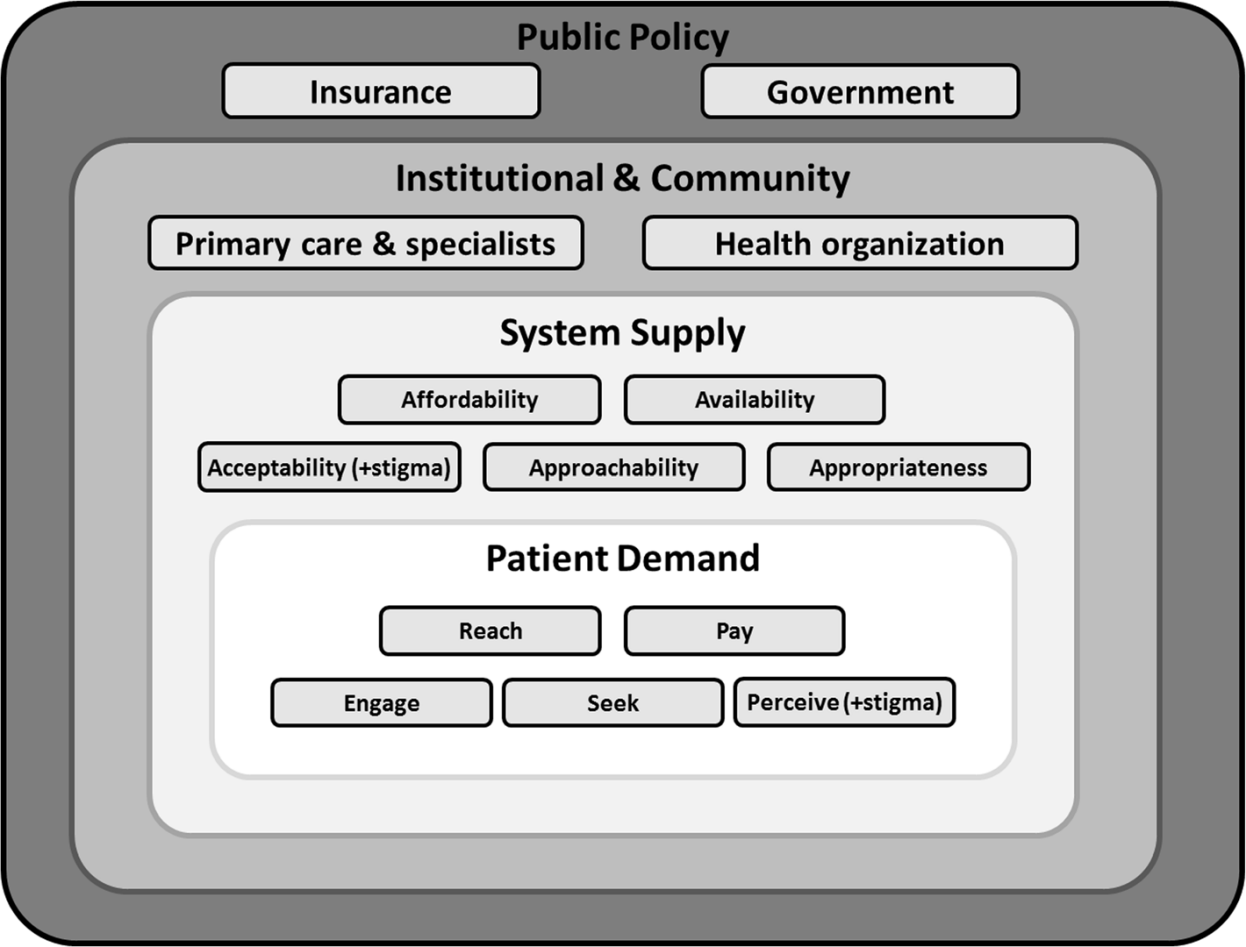
A proposed hierarchical conceptual framework for access to US urban and rural specialty care

Although our findings generally support those of Levesque et al. [22], they also illustrate that specialty care access is not a matter of a few simple issues, with multiple interconnected dynamics, some of which occur in a hierarchical manner (patients, health systems, local communities, overarching policies). In particular, the four new identified themes (government and insurance policy, health organization and operations influence, stigma, primary care and specialist influence) may occur beyond the system-supply and patient-demand dimensions in the manner portrayed by the social ecological hierarchical model [21] shown in Fig. 4, with nested patient, system, community, and policy access barriers. This adapted conceptual framework may help to further inform future research to address care access barriers.

This study has several limitations. The systematic review was limited to articles in five electronic databases published within the past five-and-a-half-years since Levesque et al.’s publication. Since only peer reviewed journal publications were considered, important findings in grey literature or conference proceedings may have been missed. Our interest in urban and rural specialty care access may have biased us towards disproportionately identifying geographic barriers. Additionally, since the geographic focus of each article was determined by its respective author(s), this may have resulted in some definition inconsistencies. While insurance is one variable that affects healthcare access, our search strategy did not include specific coverage differences; thus we were unable to identify differences between Medicaid enrollees and those with private insurance. Although a prior conceptual framework was used to organize our findings, results did not always fit clearly into one or more of its dimensions; distinguishing between system-centric (supply) and patient-centric (demand) variables also was challenging in some cases (e.g., ‘affordability’ versus ‘ability to pay’). While PRISMA guidelines do not specify screening protocols for studies, using one investigator to review titles and abstracts may marginally increase the risk of missed articles (8%) [101]. Despite our clear inclusion and exclusion criteria and our use of CMS’s definition for primary care, discerning between access to primary versus specialty care in some cases also was challenging (initial 11% inter-reader article classification discrepancy), necessitating some discussion and consensus building between reviewers [MC, AE]; both reviewers also are nurses, possibly allowing for a small professional discipline-based bias.

## Conclusions

Access to specialty care is an important and ubiquitous problem, with insufficient capacity or time delays having direct implications on health outcomes, mortality, and morbidity. As shown in the literature, causes are broad and complex, with both similarities and differences between urban and rural facilitators and barriers. Results of this systematic literature review can help researchers, policy makers, and practitioners effectively focus on important issues and needs. Since many of these interconnected issues and dynamics occur across several domains, breakthrough improvements will necessitate multi-disciplinary research that address them holistically as a system rather than individually in isolation.

## Supplementary information

**Additional file 1.** Evidence matrix summary of each reviewed publication, study design, sample size, sample characteristics, population focus (urban or rural), medical specialty foci, key findings, and methodological limitations.

**Additional file 2.** Inter-rater quality assessment results of quantitative and mixed methods studies. #1, reviewer 1; #2, reviewer 2; Av., average; Dis., inter-rater discrepancy.

## Data Availability

A summary of the papers supporting the conclusions of this article is included within the article’s additional file.

## Abbreviations

ACA: Affordable Care Act
Avail: Availability
CI: Confidence interval
CINAHL: Cumulative Index to Nursing and Allied Health Complete
CMS: Centers for Medicare and Medicaid Services
Govt: Government
HIV: Human immunodeficiency virus
MeSH: Medical subject headings
NSF: National Science Foundation
OR: Odd ratio
PCP: Primary care providers
PRISMA: Preferred Reporting Items for Systematic Reviews and Meta-Analyses
RUCA: Rural-Urban Commuting-Area
STTI: Sigma Theta Tau International
US: United States
VHA: Veterans Health Administration
