# Supplementary material for: Access to specialty healthcare in urban versus rural US populations: a systematic literature review
Source: BMC Health Serv Res. 2019 Dec 18;19:974. doi: 10.1186/s12913-019-4815-5 (PMC6921587; doi:10.1186/s12913-019-4815-5)
Supplement: Supplementary file 1 — Additional file 1. Evidence matrix summary of each reviewed publication, study design, sample size, sample characteristics, population focus (urban or rural), medical specialty foci, key findings, and methodological limitations. [file 12913_2019_4815_MOESM1_ESM.docx]

| **Reference** | **Rural, urban, both** | **Link to access framework** | **System, patient, new** | **Study design/ method** | **Sample size** | **Sample characteristics, setting** |
| --- | --- | --- | --- | --- | --- | --- |
| (Afable et al., 2018) | Both | Approachability; Appropriateness; Ability to reach | System; Patient | Mixed-methods; observational descriptive, sequential explanatory design | 7,988 e-consults (quantitative); 4 case study sites (qualitative) | VHA facilities in Connecticut, Maine, Massachusetts, New Hampshire, Rhode Island, and Vermont |
| (Allen, Davis, Hu, & Owusu-Amankwah, 2015) | Rural | Ability to pay | Patient | Quantitative; choice experiment using survey data | 796 participants | Ten Kentucky counties |
| (Bisgaier, Rhodes, & Polsky, 2014) | Urban | Affordability | System | Quantitative; audit study using secret shopper and discriminatory denials data | 273 specialty practices | 7 specialties: allergy-immunology/ pulmonary, dermatology, endocrinology, neurology, orthopedics, otolaryngology, and psychiatry in Cook County, Illinois |
| (Brooks, Dailey, Bair, & Shore, 2016) | Rural | Ability to perceive; Ability to reach | Patient | Qualitative; content analysis using focus groups | 35 female veteran participants | North Carolina, Colorado, Georgia, Hawaii, California, Washington, and Texas |
| (Bunnell, Davidson, Dewey, Price, & Ruggiero, 2017) | Both | Ability to engage | Patient | Quantitative; web-based mental health intervention with pre- & post- telephone interviews | 1,997 total (rural *n*=676; urban/suburban *n*=1,321; unable to parse out urban/suburban results) | Tornado disaster areas in Missouri and Alabama |
| (Chen, Xierali, Piwnica-Worms, & Phillips, 2013) | Rural | Gov't and insurance policy | New | Quantitative; descriptive comparison study | 760 total hospitals (456 with losses; 304 with gains) | National; based on CMS Healthcare Cost Report Information System data from 1998-2008 |
| (Chu et al., 2015) | Rural | Affordability; Appropriateness | System | Quantitative; retrospective chart review | 97 visits | Telemedicine urology patients in VHA facility in Greater (rural) Los Angeles over six-months |
| (Cummings, Wen, Ko, & Druss, 2013) | Both | Availability; Health org. | System; New | Quantitative; cross-sectional, database study, statistical modeling | 9,595 facilities; 3,141 aggregated by county for analysis | National (3,141 US counties); based on data from the 2008 National Survey of Mental Health Treatment Facilities and Area Resource File |
| (Fialkow, Snead, & Schulkin, 2017) | Both | Health org. | New | Quantitative; statistical modeling of online or paper survey-based data | 73 respondents | Practicing obstetrician-gynecologists in Washington, Wyoming, Alaska, Montana, and Idaho |
| (Flores et al., 2016) | Urban | Ability to perceive | Patient | Quantitative; cross-sectional study | 267 children | Latino and African-American children from 97 urban community sites in Dallas County, Texas |
| (Fuentes, Bjornson, Christensen, Harmon, & Apkon, 2016) | Rural | Ability to reach | Patient | Quantitative; post-intervention questionnaire and retrospective chart review | 1257 children | Comparison between White and American Indian/Alaska Native children who completed inpatient rehabilitation in the Pacific Northwest |
| (Ghali et al., 2018) | Both | Availability | System | Quantitative; retrospective review | 4,731 patients | Patients who underwent prostate cancer treatment identified within the New Hampshire State Cancer Registry (2004-2011) |
| (Grindlay, Lane, & Grossman, 2013) | Rural | Availability; Ability to perceive; Ability to reach; Stigma | System; Patient; New | Qualitative; thematic analysis of interview data | 25 patients; 15 clinical staff | Telemedicine (n=20) or in-person medical abortion services (n=5), and (n=15) clinical staff in rural Iowa clinic over 4-months |
| (Gruca, Nam, & Tracy, 2014a) | Rural | Availability; Gov't and insurance policy | System; New | Quantitative; descriptive, cross-sectional, database study | 87 otolaryngology visiting consultant clinics | Iowa otolaryngology outreach using Office of Statewide Clinical Education Programs data (2013) |
| (Gruca, Nam, & Tracy, 2014b) | Rural | Availability; Gov't and insurance policy | System; New | Quantitative; undefined, database study | 67.1 mean cities hosted a medical oncology visiting consultant clinic | Iowa oncology outreach using Office of Statewide Clinical Education Programs data (1989-2001) |
| (Gruca, Pyo, & Nelson, 2016a) | Rural | Availability | System | Quantitative; cross-sectional, database study | 167 participating cardiologists | Iowa cardiologist outreach using data from the Office of Statewide Clinical Education Programs (2014) |
| (Gruca, Pyo, & Nelson, 2016b) | Rural | Availability | System | Quantitative; cross-sectional, database study | 80 visiting orthopedic sites | Iowa orthopedic outreach using data from Iowa Physician Information System data (2014) |
| (Guerrero & Kao, 2013) | Urban | Availability | System | Quantitative; spatial autocorrelation analyses | 104 treatment facilities | National Survey of Substance Abuse Treatment Services data in Los Angeles County, California (2010) |
| (Handy et al., 2013) | Urban | Approachability; Ability to perceive; Ability to reach; Ability to pay | System; Patient | Quantitative; telephone survey | 18 patients | Uninsured patients in East Baltimore, Maryland (2009-2010) |
| (Hardy, Vivier, Rivara, & Melzer, 2013) | Both | Availability; Appropriateness; PCP/specialist influence | System; New | Quantitative; web-based or postal surveys | 433 participants | Montana pediatricians and family practice physicians (2011) |
| (Heptulla, Choi, & Belamarich, 2013) | Urban | Health org. | New | Quantitative; intervention, interrupted time series study design using statistical process control | 1 endocrine clinic (47 months of data points) | Endocrine clinic in North Bronx, New York City (2008-2011) |
| (Hine et al., 2017) | Both | Appropriateness; PCP/specialist influence | System; New | Quantitative; postal surveys | 27 physician respondents | 11 clinics (5 urban, 6 rural) in Nebraska (2011) |
| (Hirchak & Murphy, 2017) | Both | Availability | System | Quantitative; cross-sectional, database study, statistical modeling | 803 zip codes | Drug Addiction Treatment Act data in Washington State (309 rural zip codes, 494 urban, and 24 American Indian reservations) |
| (Hu, Sibert, Zhao, & Zarro, 2016) | Urban | Ability to perceive | Patient | Quantitative; cross-sectional survey study | 137 patient and 11 clinician respondents | Free clinic in Chinatown Philadelphia, Pennsylvania |
| (Kertesz et al., 2014) | Urban | Ability to reach; Ability to pay | Patient | Quantitative; cross-sectional, community-based survey | 200 respondents | Homeless population living in Birmingham, Alabama |
| (Khubchandani, Shen, Ayturk, Kiefe, & Santry, 2017) | Both | Availability | System | Quantitative; database study, geographical analysis | 2,811 emergency general surgery hospitals included | National; using data from Annual Survey of Hospitals of the American Hospital Association |
| (Kimmel et al., 2018) | Both | Availability | System | Quantitative; cross-sectional, database study, sensitivity analysis | 228 service locations | HIV comprehensive, coordinated care clinics across 16 Southern states and the District of Columbia |
| (Kozhimannil, Hung, Casey, & Lorch, 2016) | Rural | Availability | System | Quantitative; cross-sectional, database study, statistical modeling | 16,363 women | Rural residents in 9 states. Colorado, Iowa, Kentucky, New York, North Carolina, Oregon, Vermont, Washington, and Wisconsin. |
| (Kozhimannil, Hung, Henning-Smith, Casey, & Prasad, 2018) | Rural | Availability | System | Quantitative; retrospective cohort study | 4,941,387 births | National; American Hospital Association Annual Surveys and Natality Detail Data |
| (Kulcsar, Albert, Ercolano, & Mecchella, 2016) | Rural | Availability; Affordability; Appropriateness | System | Quantitative; survey, retrospective chart review, interviews | 176 patients, 244 visits | Patients who engaged in telerheumatology visits at two health system sites in Vermont and New Hampshire |
| (Larson et al., 2013) | Urban | Ability to perceive; Stigma; PCP/specialist influence | Patient; New | Quantitative; questionnaires and statistical modeling | 55 patient-child dyads | Pediatric primary care clinic in Baltimore City, Maryland |
| (Le Cook et al., 2016) | Urban | Availability; Ability to seek | System; Patient | Quantitative; secondary data analysis, statistical modeling | 12,395 respondents (4,352 Whites, 3,110 Latinos, 1,444 Asians, and 4,079 Blacks) | National; Collaborative Psychiatric Epidemiological Survey data used |
| (Li, Serban, & Swann, 2015) | Both | Availability | System | Quantitative; optimization model for spatial access | N/A | National; using data from the Cystic Fibrosis Foundation |
| (Look, Kile, Morgan, & Roberts, 2018) | Both | Availability | System | Quantitative; cross-sectional, database study, statistical modeling | 199 facilities | Wisconsin Department of Health Services and Substance Abuse Mental Health Services Administration data |
| (Lu, Plagge, Marsiglio, & Dobscha, 2016) | Urban | Appropriateness | System | Qualitative; content analysis of electronic medical records | 63 veterans | Veterans engaging in PTSD care at Portland, Oregon VHA Medical Center |
| (Maa et al., 2016) | Rural | Approachability; Affordability | System | Quantitative; case report | 1,443 veterans | Veterans who received care via the Atlanta VHA Eye Clinic Technology-based Eye Care Services program. |
| (Makaroun et al., 2017) | Urban | Health org. | New | Qualitative; telephone survey/semi-structured interviews | 18 health systems | Safety-net institutions across eight states (California, Washington, Massachusetts, Georgia, Texas, Michigan, New York, and Minnesota) |
| (Mattocks, Mengeling, Sadler, Baldor, & Bastian, 2017) | Rural | Gov't and insurance policy | New | Qualitative; semi-structured interviews | 43 VA staff and providers | Five rural VHA medical centers in the West, South, and Midwest US |
| (Mirza & Kim, 2016) | Urban | Ability to perceive | Patient | Quantitative; cross-sectional analysis of secondary survey data | 756 children from 370 households | Low-income households in a Midwestern US city |
| (Nelson et al., 2016) | Urban | Availability | System | Quantitative; prospective study | 196 consults | Teledermatology program in 11 underserved clinics, encompassing 206 dermatologic conditions in Philadelphia, Pennsylvania |
| (Nemeth et al., 2016) | Rural | Appropriateness; Ability to perceive; Ability to pay; Ability to engage | System; Patient | Qualitative; descriptive study with focus groups | 52 participants, 8 focus groups | Recent stroke survivors, their family, healthcare providers, and community leaders in low-income, Georgetown County, South Carolina |
| (Nguyen et al., 2016) | Both | Availability | System | Quantitative; cross-sectional, database, statistical modeling and sensitivity analysis | 93 counties | Counties in Nebraska using University of Nebraska Medical Center's Health Professions Tracking Service data of 2,468 behavioral health professionals |
| (Ohl et al., 2018) | Rural | Availability | System | Quantitative; cross-sectional study | 416,338 veterans | National; VHA facility Choice Act users based on VHA enrollment data |
| (Ohl et al., 2013) | Rural | Affordability; Appropriateness | System | Mixed-methods; quality improvement evaluation, feasibility study | 13 veterans interviewed; 24 under care throughout study | Veterans residing in Iowa & Illinois; Iowa City (main facility) and nine community outpatient clinics |
| (Ohl, Richardson, Kaboli, Perencevich, & Vaughan-Sarrazin, 2014) | Both | Availability | System | Quantitative; cross-sectional study | 23,669 veterans | National; Veterans Administration HIV clinical case registry data |
| (Patterson, Kaboli, Tubbs, Alexander, & Lund, 2014) | Both | PCP/specialist influence | New | Quantitative; undefined, database study, statistical modeling | 3,040,635 patients | National; Veterans Administration data |
| (Peterson, Huisingh, Girkin, Owsley, & Rhodes, 2018) | Urban | Ability to engage | Patient | Quantitative; prospective, cross-sectional study using questionnaire data | 110 patients | Glaucoma clinic at University of Alabama Birmingham |
| (Pinto, Rochat, Hennink, Zertuche, & Spelke, 2016) | Rural | Affordability; Appropriateness; Gov't and insurance policy; Stigma; PCP/ specialist influence | System; New | Qualitative; semi-structured interviews | 46 participants | Sampled from obstetricians, maternal-fetal medicine specialists, certified nurse midwives, and maternal and infant health leaders in Atlanta, Georgia |
| (Powell et al., 2018) | Urban | Health org.; Stigma | New | Qualitative; content analysis | 15 general nonprofit hospitals | Facilities in Philadelphia County (and adjacent Bucks, Chester, Delaware, and Montgomery Counties) that had community health needs assessment data |
| (Price, Sewell, Chen, & Sarkar, 2016) | Urban | PCP/specialist influence | New | Quantitative; descriptive study | 1,304 included referrals | Gastroenterology clinic referrals at Zuckerberg San Francisco General safety-net hospital in San Francisco, California |
| (Ramanuj, Talley, Breslau, Wang, & Pincus, 2018) | Urban | Appropriateness; Gov't and insurance policy; Health org.; PCP/specialist influence | System; New | Qualitative; semi-structured interviews | 52 interview participants (36 senior clinicians and administrators; 16 individual with frontline staff) | 11 behavioral health-primary care integration sites in New York City |
