# Supplementary material for: Access to specialty healthcare in urban versus rural US populations: a systematic literature review
Source: BMC Health Serv Res. 2019 Dec 18;19:974. doi: 10.1186/s12913-019-4815-5 (PMC6921587; doi:10.1186/s12913-019-4815-5)
Supplement: Supplementary file 2 — Additional file 2. Inter-rater quality assessment results of quantitative and mixed methods studies. #1, reviewer 1; #2, reviewer 2; Av., average; Dis., inter-rater discrepancy. [file 12913_2019_4815_MOESM2_ESM.docx]

| Quantitative | #1 | #2 | Dis. | Av. | Quantitative | #1 | #2 | Dis. | Av. |
| --- | --- | --- | --- | --- | --- | --- | --- | --- | --- |
| (Afable et al., 2018) | 0.75 | 0.75 | 0.14 | 0.75 | (Kulcsar, et al., 2016) | 0.85 | 0.80 | 0.21 | 0.83 |
| (Allen, et al., 2015) | 1.00 | 0.91 | 0.07 | 0.95 | (Larson et al., 2013) | 0.86 | 0.82 | 0.07 | 0.84 |
| (Bisgaier, et al., 2014) | 0.91 | 0.82 | 0.29 | 0.86 | (Le Cook et al., 2016) | 1.00 | 1.00 | 0.00 | 1.00 |
| (Bunnell, et al., 2017) | 0.79 | 0.79 | 0.14 | 0.79 | (Li, et al., 2015) | 0.81 | 0.81 | 0.00 | 0.81 |
| (Chen, et al., 2013) | 0.65 | 0.90 | 0.36 | 0.78 | (Look, et al., 2018) | 0.94 | 1.00 | 0.07 | 0.97 |
| (Chu et al., 2015) | 0.60 | 0.75 | 0.21 | 0.68 | (Maa et al., 2016) | 0.67 | 0.67 | 0.00 | 0.67 |
| (Cummings, et al., 2013) | 1.00 | 1.00 | 0.00 | 1.00 | (Mirza & Kim, 2016) | 0.95 | 1.00 | 0.07 | 0.98 |
| (Fialkow, et al., 2017) | 0.90 | 0.85 | 0.07 | 0.88 | (Nelson et al., 2016) | 1.00 | 0.94 | 0.07 | 0.97 |
| (Flores et al., 2016) | 1.00 | 1.00 | 0.00 | 1.00 | (Nguyen et al., 2016) | 0.95 | 0.91 | 0.07 | 0.93 |
| (Fuentes, et al., 2016) | 0.91 | 0.86 | 0.14 | 0.89 | (Ohl et al., 2018) | 0.85 | 0.90 | 0.07 | 0.88 |
| (Ghali et al., 2018) | 0.90 | 1.00 | 0.14 | 0.95 | (Ohl et al., 2013) | 0.77 | 0.77 | 0.00 | 0.77 |
| (Gruca, et al., 2014a) | 0.65 | 0.65 | 0.00 | 0.65 | (Ohl, et al., 2014) | 1.00 | 0.91 | 0.07 | 0.95 |
| (Gruca, et al., 2014b) | 0.61 | 0.78 | 0.21 | 0.69 | (Patterson, et al., 2014) | 1.00 | 1.00 | 0.00 | 1.00 |
| (Gruca, et al., 2016a) | 0.60 | 0.65 | 0.14 | 0.63 | (Peterson, et al., 2018) | 1.00 | 1.00 | 0.00 | 1.00 |
| (Gruca, et al., 2016b) | 0.85 | 0.85 | 0.00 | 0.85 | (Price, et al., 2016) | 0.78 | 0.78 | 0.00 | 0.78 |
| (Guerrero & Kao, 2013) | 1.00 | 1.00 | 0.00 | 1.00 | (Rauh, et al., 2018) | 1.00 | 1.00 | 0.00 | 1.00 |
| (Handy et al., 2013) | 0.73 | 0.73 | 0.00 | 0.73 | (Slivinski, et al., 2017) | 0.75 | 0.50 | 0.14 | 0.63 |
| (Hardy, et al., 2013) | 0.82 | 0.82 | 0.00 | 0.82 | (Smulian et al., 2016) | 0.83 | 0.83 | 0.00 | 0.83 |
| (Heptulla, et al., 2013) | 0.77 | 0.77 | 0.00 | 0.77 | (Southard, et al., 2014) | 0.80 | 0.95 | 0.21 | 0.88 |
| (Hine et al., 2017) | 0.50 | 0.63 | 0.21 | 0.57 | (Tan et al., 2018) | 0.86 | 0.86 | 0.00 | 0.86 |
| (Hirchak & Murphy, 2017) | 0.95 | 0.95 | 0.00 | 0.95 | (Temkin, et al., 2015) | 0.91 | 1.00 | 0.14 | 0.95 |
| (Hu, et al., 2016) | 0.90 | 0.90 | 0.14 | 0.90 | (Turner et al., 2013) | 1.00 | 1.00 | 0.00 | 1.00 |
| (Kertesz et al., 2014) | 0.95 | 0.95 | 0.00 | 0.95 | (Vick, 2016) | 1.00 | 1.00 | 0.00 | 1.00 |
| (Khubchandani, et al., 2017) | 0.82 | 0.86 | 0.07 | 0.84 | (Ward et al., 2014) | 0.59 | 0.77 | 0.29 | 0.68 |
| (Kimmel et al., 2018) | 0.64 | 0.73 | 0.14 | 0.68 | (Williams et al., 2013) | 0.70 | 0.75 | 0.21 | 0.73 |
| (Kozhimannil, et al., 2016) | 0.95 | 1.00 | 0.07 | 0.98 | (Wright, et al., 2016) | 0.85 | 0.85 | 0.14 | 0.85 |
| (Kozhimannil, et al., 2018) | 1.00 | 1.00 | 0.00 | 1.00 | (Yan et al., 2013) | 0.91 | 1.00 | 0.14 | 0.95 |
| Qualitative | #1 | #2 | Dis. | Av. | Qualitative | #1 | #2 | Dis. | Av. |
| (Afable et al., 2018) | 0.65 | 0.70 | 0.10 | 0.68 | (Powell et al., 2018) | 0.90 | 0.90 | 0.20 | 0.90 |
| (Brooks, et al., 2016) | 0.80 | 0.80 | 0.00 | 0.80 | (Ramanuj, et al., 2018) | 1.00 | 1.00 | 0.00 | 1.00 |
| (Grindlay, et al., 2013) | 0.70 | 0.80 | 0.20 | 0.75 | (Ray, et al., 2015) | 0.80 | 0.90 | 0.10 | 0.85 |
| (Lu, Plagge, et al., 2016) | 0.95 | 1.00 | 0.10 | 0.98 | (Smulian et al., 2016) | 0.65 | 0.75 | 0.20 | 0.70 |
| (Makaroun et al., 2017) | 0.90 | 0.90 | 0.00 | 0.90 | (Vermette, et al., 2015) | 0.85 | 0.85 | 0.00 | 0.85 |
| (Mattocks, et al., 2017) | 0.75 | 0.90 | 0.20 | 0.83 | (Warner, et al, 2014) | .85 | 0.90 | 0.10 | 0.88 |
| (Nemeth et al., 2016) | 1.00 | 1.00 | 0.00 | 1.00 | (Ziemer et al., 2014) | 0.80 | 0.90 | 0.20 | 0..85 |
| (Pinto, et al., 2016) | 0.90 | 0.90 | 0.00 | 0.90 |  |  |  |  |  |
| *Table S2.* Inter-rater quality assessment results of quantitative and mixed methods studies. #1, reviewer 1; #2, reviewer 2; Av., average; Dis., inter-rater discrepancy. | | | | | | | | | |
